# Decision making in frontotemporal dementia: neuropsychological and neuroimaging assessments

**DOI:** 10.3389/fnins.2025.1632054

**Published:** 2025-07-31

**Authors:** Shima Tavakolan Haghighi, Chiara Bertasini, Rozalda Picari, Giovanna Paolone

**Affiliations:** Department of Diagnostic and Public Health, Pharmacology Section, University of Verona, Verona, Italy

**Keywords:** frontotemporal dementia, decision-making tasks, cognitive deficits, neuroimaging studies, brain lesions

## Abstract

Frontotemporal dementia (FTD) is a neurodegenerative disorder primarily affecting the frontal and temporal lobes, leading to significant impairments in executive function, social behavior, and emotional regulation. FTD is clinically categorized into two variants, the behavioral (bvFTD) and the primary progressive aphasia (PPA). The bvFTD is the most common form of FTD, and, in these patients, impaired decision-making is the most prominent and clinically relevant cognitive deficit, often manifesting early and preceding more global cognitive decline. This narrative review explores the neuropsychological and neurobiological basis of decision-making deficits in FTD, with a focus on tasks such as the Moral Behavior Inventory (MBI), Iowa Gambling Task (IGT), Executive and Social Cognition Battery (ESCB), Addenbrooke’s Cognitive Examination (ACE), and the Balloon Analog Risk Task (BART). Evidence from both case and cohort studies reveals that, although bvFTD patients have intact cognitive profiles, they show impaired performance on decision-making tasks, suggesting that these deficits may be an early and specific marker of the disease. In addition, according to neuroimaging studies, these impairments are associated with atrophy in a distributed network, including the ventromedial and orbitofrontal cortices, anterior cingulate, insula, and even cerebellar regions. Finally, impaired decision-making is a critical yet underrecognized marker for early detection and differentiation of FTD from other forms of dementia.

## 1 Introduction

Frontotemporal dementia (FTD) is a neurodegenerative disorder that progressively leads to the neurodegeneration of the frontal and temporal lobes of the brain. The global prevalence of FTD is estimated at 15–22 cases per 100,000 individuals, with an annual incidence of approximately 2.7–4.1 per 100,000, making FTD the second most common etiology of early-onset dementia in individuals younger than 65 years (Harvey, 2003; Onyike and Diehl-Schmid, 2013). FTD has a median survival of 3–7 years post-symptom onset, and its clinical phenotype is notably heterogeneous, reflecting a broad spectrum of cognitive, behavioral, and language impairments that vary substantially across affected individuals (Rankin, 2020). Particularly, FTD patients are characterized by behavioral and personality changes, including loss of insight, deterioration of social interactions, difficulties in regulating personal conduct, perseveration, stereotyped behaviors, and disinhibition (Elfgren et al., 1993; Gustafson, 1993; Neary et al., 1988; Cosseddu et al., 2020). FTD is clinically categorized into two primary syndromic variants. The most common is the behavioral variant (bvFTD), also referred to as the frontal variant FTD (fvFTD; Diehl-Schmid et al., 2011), which accounts for approximately 60% of FTD cases. This form is characterized by prominent behavioral dysregulation, significant changes in personality, social behavior, and executive functioning (Johnson et al., 2005). The second major subtype is the primary progressive aphasia (PPA), which includes the semantic-variant (svPPA), also known as semantic dementia (SD), the nonfluent-variant (nfvPPA), and the logopenic variant (lvPPA; Gorno-Tempini et al., 2011). These forms of FTD predominantly impair speech and language abilities (McKhann, 2001), although behavioral alterations have also been reported (Perry et al., 2006). In addition, a temporally predominant subtype (temporal variant FTD, tvFTD) and a right temporal variant of FTD (rtvFTD) have also been observed, associated with SD (Perry et al., 2006), leading to deficits in semantic knowledge, language comprehension, behavioral abnormalities, and emotional processing impairments (Perry and Hodges, 2000; Liu et al., 2004). A schematic classification of the FTD variants is reported in Table 1.

**Table 1:** Schematic classification of the FTD variants.

FTD variant classification	Symptoms and clinical features			Anatomical correlates
bvFTD (or fvFTD)	Variant with cognitive and behavioral impairments such as personality changes, loss of empathy, disinhibition, apathy, impulsivity, loss of insight, eating disorders, stereotyped behavior			Atrophy in frontal and temporal lobes, in insula and anterior cingulate cortex
PPA	Progressive decline in linguistic skills, language deficits, difficulty in speech, subdivided into:	svPPA (or SD)	Involves mainly left temporal lobe. Impaired word comprehension and naming (anomia), impaired object knowledge, dyslexia/dysghraphia, emotional withdrawal	Asymmetric atrophy of anterior temporal lobe
		nfvPPA	Grammar misuse (agrammatism), effortful and halting speech production, apraxia, impaired sentence comprehension	Atrophy of the left inferior frontal/insular cortex
		lvPPA	Impaired single-word retrieval, impaired sentence repetition, phonologic errors	Asymmetric atrophy of left posterior temporal, inferior parietal lobe and medial temporal lobe
tvFTD	FTD variant with predominant temporal lobes atrophy, behavioral and language impairments	rtvFTD	Involves right temporal lobe. Prosopagnosia, memory deficits, behavioral impairments, e.g., apathy, disinhibition, compulsiviness	Bilateral asymmetrical atrophy in anterior temporal lobes and right ventral frontal area

The schematic table below summarizes the classifications of FTD variants, associated symptoms, clinical features, and their anatomical correlations (Seeley et al., 2008; Ranasinghe et al., 2016).

The social and behavioral changes observed in FTD variants have been associated with three main neurocognitive frameworks, such as the controlled social-semantic cognition (CS-SC) model (Rouse et al., 2024), the transdiagnostic spectrum approach (Rohrer et al., 2015), and the network degeneration model (Seeley et al., 2009), with the CS-SC model emerging as the most dominant framework. According to this model, the social and behavioral alterations are related to impairments in the anterior temporal lobes (ATL) and prefrontal cortex (PFC) regions. Specifically, SD behavioral changes are due to ATL atrophy resulting in loss of semantic-social knowledge, while impaired social control from PFC primarily poses bvFTD behavioral changes (Rouse et al., 2024).

The transdiagnostic spectrum approach considers FTD as a continuous multidimensional spectrum of symptoms due to the overlapping of bvFTD, svPPA, nfvPPA, lvPPA clinical features and brain atrophy patterns (Rohrer et al., 2015).

Finally, the network degeneration model described by Seeley et al. (2009) defines FTD as a large-scale, targeted and spreading neurodegenerative process among specific brain networks.

Impaired decision-making is a hallmark cognitive deficit in FTD, particularly in bvFTD. These impairments are primarily driven by neurodegeneration in prefrontal regions, especially the ventromedial and orbitofrontal cortices, which are essential for evaluating risk–reward contingencies, processing social–emotional cues, and guiding goal-directed behavior (Rahman et al., 1999; Bechara et al., 2000). Furthermore, compulsivity is a common feature in bvFTD, marked by repetitive and ritualistic behaviors such as hoarding, excessive cleaning, and verbal repetition. These actions, often lacking practical value, are linked to dysfunction in the orbitofrontal and anterior cingulate cortices. Patients typically show rigid thinking and difficulty adapting to changes, with some exhibiting hypersexuality, overeating, or gambling. These behaviors are believed to stem from disrupted fronto-subcortical circuits involved in the regulation of motivation and impulse control (Moheb et al., 2019).

Clinically, patients affected by bvFTD often exhibit impulsivity, impaired judgment, and a diminished capacity to anticipate consequences, resulting in socially inappropriate actions and maladaptive real-life decisions, such as financial mismanagement (Torralva et al., 2007). These symptoms reflect the disruption of prefrontal networks involved in value-based judgment and social cognition. Given the substantial impact of compromised decision-making on the daily lives of both patients and their caregivers, numerous studies have focused on investigating this crucial aspect of FTD.

Moreover, a variety of neuropsychological tasks have been employed to assess moral reasoning, risk-taking, and reward evaluation. Among the most used tools are the Moral Behavior Inventory (MBI), the Iowa Gambling Task (IGT), the Executive and Social Cognition Battery (ESCB), the Addenbrooke’s Cognitive Examination (ACE), and the Balloon Analog Risk Task (BART; Bechara et al., 1994; Lejuez et al., 2002; Mendez et al., 2005b; Mendez et al., 2005a; Kloeters et al., 2013).

Furthermore, bvFTD patients are frequently misdiagnosed with psychiatric disorders or neurological syndromes, such as major depression or Alzheimer’s disease (AD; Bertoux et al., 2013; Custodio et al., 2021) due to overlapping in both clinical symptoms and impaired cognitive domains (Forman et al., 2006). To improve the diagnostic precision of FTD and effectively distinguish FTD from AD, several neuropsychological assessment tools for evaluating social and emotional cognition have been proposed, including the Social Cognition and Emotional Assessment (SEA) and its brief version Mini-SEA (Custodio et al., 2021), the Faux Pas Test, the Awareness of Social Inference Test (TASIT), and the Dynamic Affect Recognition Test (DART; Rankin et al., 2024).

In this narrative review, we sought to explore the alterations in decision-making observed in patients with FTD by describing each tool separately to highlight their specific contribution; however, in clinical practice, they are often used in parallel to capture complementary aspects of cognitive and behavioral dysfunction in FTD. Furthermore, most of the studies associated the evaluation of decision-making behavior with neuroimaging analyses to better understand the underlying neural correlates (Kipps et al., 2008).

## 2 Methods

Articles relevant to the aim of this review were selected by applying a range of targeted search strings on PubMed and Google Scholar. These included:

1. *(“Frontotemporal dementia” OR “bvFTD”) AND (“decision-making” OR “risky behavior” OR “impulsivity”) AND (“neuropsychological assessment” OR “cognitive tests”)*,
2. *(“Frontotemporal dementia” OR “bvFTD”) AND (“Salience Network” OR “Default Mode Network” OR “Semantic Appraisal Network”) AND (“fMRI” OR “connectivity” OR “neuroimaging”)*,
3. *(“Frontotemporal dementia” OR “bvFTD”) AND (“social cognition” OR “emotion recognition” OR “theory of mind”) AND (“SEA” OR “Mini-SEA” OR “TASIT” OR “Faux Pas Test”)*, and
4. *(“bvFTD” OR “behavioral* var*iant frontotemporal dementia”) AND* (*“early diagnosis” OR “differential diagnosis”*) *AND (“Alzheimer’s disease” OR “psychiatric disorders”) AND (“neuropsychological tools” OR “executive functions”) (“primary progressive aphasia” or “PPA”) AND (“semantic variant FTD” OR “semantic dementia” OR “non fluent variant FTD”).*

## 3 Neuropsychological tests for FTD diagnosis

Assessing decision-making in patients with FTD is crucial for understanding their capacity for everyday choices, financial management, and long-term planning. A wide range of neuropsychological tests have been employed across studies to evaluate this impairment, each targeting specific aspects of decision-making and executive function (Table 2).

**Table 2:** Decision-making assessment in FTD patient: behavioral and neuroimaging investigations.

Study	Behavioral results	Neuroimaging investigations
Pathological gambling in FTD (Coco and Nacci, 2004)	BvFTD patient presented with pathological gambling; Frontal lobe impairment revealed by neuropsychological tests.	Brain MRI: frontal and temporal atrophy.
“Trolley problem” study (Fong et al., 2017)	bvFTD patients show faster and apathic decision-making capability	n/a
Decision-making in PPA (Gleichgerrcht et al., 2012)	“Flat” decision-making profile of PPA patients during IGT.	n/a
Gambling decision and atrophy in bvFTD (Kloeters et al., 2013)	Altered cognitive functions (ACE) of bvFTD patients; Altered modified total net score of IGT showed by bvFTD patients. Conflicting results from previous IGT studies.	IGT modified total net score of bvFTD patients: atrophy in the PFC, occipital cortex, and cerebellum.
Decision-making implications in FTD (Manes et al., 2010, 2011; Manes et al., 2011)	Compromised IGT performance of both NNP and INP groups, specifically in last 3 blocks.	n/a
FTD case study (Manes et al., 2010)	Altered IGT performance of bvFTD patient.	MRI scan: progressive atrophy of the frontal lobe; HMPAO-SPECT: hypoperfusion of frontal and temporal lobes.
Decision-making assessment using MBI (Mendez et al., 2005a)	FTD patients show greater impairments in executive functions than AD patients. Awareness of right or wrong is not impaired in FTD patients.	n/a
bvFTD presenting with pathological gambling (Nakaaki et al., 2007)	Case report of bvFTD: altered IGT performance; Preserved executive function tasks, memory and visuospatial functions.	Brain MRI: bilateral mild frontal lobe atrophy; 99mTc-ECD-SPECT: bilateral hypoperfusion in the frontal lobes.
Deficits in bvFTD (Rahman et al., 1999)	bvFTD patients showed pronounced risk-taking behavior in a decision-making paradigm.	n/a
Decision-making difficulties in a linguistic context (Spotorno et al., 2015)	bvFTD patients exhibit decision-making limitations in case of ambiguous anaphoric pronouns.	high-resolution volumetric T1-weighted MRIs: atrophy in medial and orbital frontal regions and in the right insula.
Decision-making assessment with BART (Strenziok et al., 2011)	Impaired BART performance of bvFTD patients.	n/a
Decision-making in bvFTD (Torralva et al., 2007)	Impaired IGT performance of bvFTD patients.	n/a
Different battery of tests for FTD (Torralva et al., 2009)	bvFTD patients: hiACE and loACE; loACE: impaired performance across ESCB; hiACE: altered IGT performane in block 3, 4, and 5.	n/a

### 3.1 Moral behavior inventory

The MBI test is a questionnaire designed to minimize cultural and religious biases while maximizing the content validity of empathy and fairness. The participants are asked to rate various behaviors on a 4-point scale as “not wrong,” “mildly wrong,” “moderately wrong,” or “severely wrong” (Mendez et al., 2005a). FTD patients are often evaluated compared to patients affected by other forms of dementia, such as AD, to serve as a clinical control group and to help isolate cognitive and behavioral deficits specific to FTD (Mendez et al., 2005a). Overall, FTD patients show greater impairments in executive functions, while AD patients perform worse in memory tasks. Notably, according to the MBI, patients with FTD did not show significant impairment in their awareness of right or wrong principles compared to patients with AD or control individuals. Indeed, despite the intact capability of FTD patients to understand moral rules, they show a reduced emotional identification with other people, and they usually solve moral dilemmas without emotional involvement (Mendez et al., 2005a). In agreement with this study, to assess moral decision-making, particularly in participants with emotionally charged ethical dilemmas, Fong et al. (2017) exploited the classic “trolley problem” task of which two versions are mostly used: an impersonal dilemma, where one must flip a switch to divert a runaway trolley, sacrificing one life to save five; and a personal dilemma, where stopping the trolley requires directly pushing a person off a bridge, an action that involves intentional physical harm. These scenarios are designed to probe the interplay between cognitive reasoning and emotional inhibition. In this study, bvFTD patients were compared to AD patients and healthy controls. While all groups responded similarly to the impersonal scenario, 90% of bvFTD patients endorsed the personal dilemma, compared to only 45% of both AD patients and controls. Moreover, bvFTD patients made these decisions faster and showed less emotional discomfort in their self-reports, exhibiting less consideration for the repercussions of their actions (Fong et al., 2017). This is not so surprising since FTD patients often show sociopathic and antisocial behavior, including undesired sexual acts, traffic violations, stealing, and physical assaults (Mendez et al., 2005b).

### 3.2 Iowa gambling task

The IGT test has been used in numerous studies to demonstrate significant impairments in the decision-making capability, even in the early stages of the disease (Mendez et al., 2005a).

IGT is usually part of a specific battery of tests designed to detect the core deficits characteristic of bvFTD. Consequently, evaluations that replicate real-life scenarios encompassing decision-making and social interactions are employed to assess these functions. While performing the IGT, individuals have to choose cards from 4 different decks (A, B, C, or D). Typically, decks A and B represent the disadvantageous selection, as they offer high immediate rewards but are also associated with occasional significant losses, ultimately leading to a net loss over time. Decks C and D represent the advantageous selection, providing smaller immediate rewards accompanied by only minor losses, resulting in a cumulative gain in the long term (Bechara et al., 1994). This test is used to evaluate decision-making processes, and its link to pathological gambling is far from coincidental, as it is a behavioral alteration that often characterizes bvFTD-affected patients. This condition emerges as an early indicator of possible underlying cognitive dysfunctions, especially when it appears in people with no history of prior addictions or when it cannot be attributed to substance abuse. In 2004, Coco and Nacci reported for the first time a bvFTD case affected by gambling addiction. Neuropsychological assessments demonstrated marked deficits indicative of frontal lobe dysfunction, while magnetic resonance imaging (MRI) confirmed atrophy in the frontal and temporal regions. These combined findings supported the clinical diagnosis of bvFTD (Coco and Nacci, 2004).

One of the earliest applications of the IGT was to assess its sensitivity in detecting cognitive impairments in individuals with early or mild bvFTD. Results clearly reported the preference of bvFTD patients to consistently choose risky and disadvantageous decks, displaying deficits in decision-making when compared to control subjects (Torralva et al., 2007).

Similarly, Nakaaki et al. (2007) presented a case report of a patient with mild bvFTD who exhibited abnormal behavior, including altered sexual behavior and pathological gambling. Moreover, this case study showed poor IGT performance, which was partly attributed to an impaired capacity of the patient to foresee future consequences. Indeed, unlike control individuals, the subject showed a marked preference for disadvantageous choices throughout the entire duration of the task (Nakaaki et al., 2007).

These findings highlight that decision-making impairments emerge early during FTD, thus reinforcing the clinical utility of the IGT as a sensitive tool for early diagnosis. This pattern becomes more evident when, based on their cognitive profiles, bvFTD patients were divided into two groups: those with normal neuropsychological performance (NNP) and those with impairments (INP). As expected, the INP group performed significantly worse than both the NNP group and the control group on most cognitive measures, while the NNP group showed no significant differences from controls across standard neuropsychological tests. In contrast, decision-making performance assessed through the IGT showed different results. In the final blocks of the task, when learning should guide better choices, both patient groups performed significantly worse than controls, despite not differing from each other. This suggests that even bvFTD patients who appear cognitively intact on traditional tests may have profound impairments in real-world decision-making (Manes et al., 2011).

Additionally, the clinical and diagnostic application of IGT was confirmed by a case report described by Manes et al. (2011), who exhibited alterations exclusively in the IGT with performance patterns resembling those typically observed in bvFTD. Importantly, this deficit preceded a marked cognitive and behavioral decline over the following 12 months. The bvFTD diagnosis was then confirmed by the MRI scan showing a progressive atrophy of the frontal lobe, and by the marked hypoperfusion of frontal and temporal lobes assessed through hexamethylpropyleneamine oxime single photon emission CT (HMPAO-SPECT). In addition, the patient manifested the typical phenotypic profile of bvFTD, followed, 2 years later, by a marked decline in performance across multiple tasks assessing executive functions (Manes et al., 2010).

Finally, the IGT has also been employed to evaluate decision-making in patients with PPA. The results indicated that, unlike bvFTD patients, who typically show impulsive and risky decision-making, PPA patients did not display this pattern as their performance on the test was neutral, meaning they neither learned from the task nor behaved impulsively. While they fully understood the task instructions, subtle language deficits may have indirectly influenced their engagement or decision-making strategies (Gleichgerrcht et al., 2012). Clearly, bvFTD patients experience compromised decision-making processing that could interfere with language processing. When involved in a study on ambiguous anaphoric pronouns requiring cognitive flexibility, participants were asked to determine which noun a given pronoun referred to within a sentence, bvFTD performed normally when the reference was clear or indirectly inferable, while in truly ambiguous situations, they significantly underperformed compared to healthy controls (Spotorno et al., 2015).

### 3.3 Executive and social cognition battery and Addenbrooke’s cognitive examination

The ESCB and the ACE tests explore separate, although complementary, aspects of cognitive impairments in FTD, therefore, they are often administered together.

The ESCB is a specific neuropsychological tool developed to evaluate both executive functions and social cognition, which has been proven to have a greater discriminatory power in distinguishing between bvFTD patients and healthy controls than traditional executive function tests. This test focuses on detecting impairments in key areas such as planning, decision-making, and social cues understanding; furthermore, by including tasks that mimic real-life situations, ESCB is particularly suitable to identify subtle deficits (Torralva et al., 2009). Instead, the ACE, a commonly used test created at the Addenbrooke’s Hospital in Cambridge, United Kingdom [undergone two revisions: ACE-Revised and the current ACE-Third Edition (III)], offers a broad overview of global cognitive function. When administered together, these tools provide a more comprehensive assessment of cognitive status in bvFTD, particularly in the early stages of the disease (Torralva et al., 2009). The ACE evaluates five major domains of cognitive functioning: attention and orientation, memory, verbal fluency, language, and visuospatial abilities, has been proven effective in monitoring disease progression in FTD, offering a practical and non-invasive means of tracking cognitive decline without the need for longitudinal neuroimaging data (Kipps et al., 2008). The full version of the ACE yields a score out of 100, with sub-scores for each domain, providing clinicians with a nuanced profile of the subject’s cognitive strengths and weaknesses. Administration typically requires 15–20 min, making it practical for use in outpatient and primary care settings (Mioshi et al., 2006). According to the dementia cut-off score, patients are clustered in high-functioning (hiACE) and low-functioning (loACE) groups (Rahman et al., 1999).

According to Torralva et al. (2009), the loACE group showed significant impairments across all measures of the ESCB compared to healthy controls. Similarly, the hiACE group, despite overall better cognitive performance, still showed impairments relative to controls on tasks evaluating real-life planning and organization, theory of mind (ToM, the ability to attribute mental states to others), and decision-making. In the same study, to capture different dimensions of cognitive dysfunctions, patients were evaluated with both ACE and IGT. Accordingly, the hiACE patients exhibited a severe impairment during blocks 3, 4, and 5 of the IGT. However, Kloeters et al. (2013) reported slightly different results as the performance of hiACE patients in IGT was similar to controls, in fact some patients improved over time, gradually shifting toward more advantageous deck selections. This unexpected finding was attributed to substantial variability in performance, where some bvFTD patients behaved similarly to controls, and some controls exhibited atypical or inconsistent decision-making patterns (Kloeters et al., 2013).

### 3.4 Balloon analog risk task

The BART has also been used to investigate impaired decision-making in bvFTD, offering insight into patients’ risk-taking behavior under conditions of uncertainty. This task is used to assess risk-taking behavior under laboratory conditions. Through a computer simulation, individuals have to pump to inflate balloons until they explode or until the patient decides to end the trial to obtain the monetary reward. Each pump indeed corresponds to a specific reward (Lejuez et al., 2002). bvFTD patients showed significantly worse performance on BART compared to control individuals, reporting a reduced number of pumps with no signs of progressive learning. Indeed, patients were more prone to suspend pumping to obtain their reward earlier, not being willing to take the risk of reaching a higher monetary gain. Conversely, controls tried to maximize their gains by increasing the number of pumps, explored the expansion of the balloon before the explosion, and showed increased learning throughout the task, leading to modifications in their behavior according to past information (Strenziok et al., 2011).

## 4 Neuropsychological assessment of social cognition and decision-making in FTD

Deficit in social cognition contributes significantly to the impaired decision-making in FTD patients, particularly in the social and moral context. In light of this, several useful neuropsychological tools have been indicated in literature to assess social and emotional domains in bvFTD patients. A brief overview of these tools is presented.

The Social Cognition and Emotional Assessment (SEA) battery test has been exploited to evaluate facial emotion recognition and ToM deficits, which are hallmark features in bvFTD patients. Moreover, this test examines cognitive dysfunction, specifically motivation, behavioral control, and reversal learning impairments (Bertoux et al., 2014).

The Mini-SEA, a short version of SEA, comprises the Faux Pas test, which assess the ToM by describing different social situations and evaluate patient’s ability to detect the socially inappropriate remarks (Faux Pas), and the Facial Emotion Recognition test, which evaluates patient’s ability to identify and interpret facial emotional expressions (Custodio et al., 2021).

Evaluation of behavioral deficits is generally assessed by clinical scales such as Cambridge Behavioral Inventory (CBI) or Neuropsychiatric Inventory (NPI; Bertoux et al., 2014). The CBI is an 81-item questionnaire aimed to evaluate memory, orientation and attention, everyday skills, self-care, mood and beliefs, challenging and stereotypic behaviors, disinhibition, eating habits, sleep, motivation and insight which result compromised in bvFTD, AD, Parkinson disease (PD) and Huntington’s disease (HD; Wedderburn et al., 2008). Particularly, CBI highlights a bvFTD profile encompassing behavioral deficits with motivation and stereotypic behavior impairments (Wedderburn et al., 2008). In addition, the Frontal Behavioral Inventory (FBI), the Interpersonal Reactivity Index- Emphatic Concern (IRI-EC), the IRI-perspective taking (IRI-PT), and the Revised Self-Monitoring Scale (r-SMS) are also effectively employed in clinical settings to assess behavioral abnormalities in bvFTD patients (Custodio et al., 2021).

The FBI is an informant-based behavioral questionnaire created to identify bvFTD according to the intensity of the symptoms. It gives a score ranging from 0 to 72 to positive or negative symptoms, such as aggression, apathy, loss of insight, in which the highest score indicates severe disturbances (Custodio et al., 2021).

Instead, the IRI measures the tendency of patients to identify with fictional characters, their willingness to adopt the perspective of other people (IRI-PT), their capacity to experience concerns for others (IRI-EC), and even their tendency to feel personal discomfort in response to others’ negative experiences.

The r-SMS is another questionnaire made up of 13 items aiming to measure the ability of patients to adapt their behavior to a particular social context and their sensitivity to emotional cues during face-to-face interactions (Custodio et al., 2021). It has also been reported by Custodio and colleagues that FBI, IRI-EC and IRI-PT tests are valuable tools to distinguish bvFTD from AD, while the application of mini-SEA with ACE-III showed higher sensitivity and specificity to differentiate bvFTD and AD patients.

Another neuropsychological test to assess social cognition, emotion recognition, and understanding of sarcasm or deception is the TASIT, used in patients with traumatic brain injury and neurodegenerative conditions such as FTD, AD, PD, Amyotrophic Lateral Sclerosis (ALS), and FTD-ALS (McDonald et al., 2003; Multani et al., 2019; Savage et al., 2013). Additionally, DART is a freely available, digitally administered 12-item emotion labeling task designed to assess emotion identification deficits in cognitively impaired patients (Rankin et al., 2024).

Finally, the Uniformed Data Set-Frontotemporal Lobar degeneration (UDS-FTLD) battery represents an informative clinical tool that discriminates between bvFTD and PPA variants (svPPA, nfvPPA, logopenic PPA; Staffaroni et al., 2021). The UDS-FTLD assessment includes a range of key language tasks (e.g., word reading, semantic matching, anagram, and sentence repetition), and behavior measures, such as, social behavior observer checklist, IRI informant questionnaire, and r-SMS informant questionnaire (Weintraub et al., 2018; Gefen et al., 2020). This battery allows differential and highly sensitive diagnosis for FTD variants but also helps in monitoring disease progression and behavioral symptoms. Although it has clinical approach, UDS-FTLD battery does not cover cognitive aspects as it lacks tests for visual perception or complex executive functions. Moreover, individuals with reduced insight, such as bvFTD patients, generate unreliable self-report questionnaires (Gefen et al., 2020).

Given the complexity of the cognitive profiles in bvFTD patients compared to AD and other forms of dementia, it is fundamental to acknowledge that cognitive deficit patterns change across disease progression. A large cohort study performed by Ranasinghe et al. (2016) compared bvFTD neuropsychological and neuropsychiatric symptoms with an age-matched group of AD patients applying the Clinical Dementia Rating (CDR) scale, which measures the different stages of the disease. The results indicated that in the very early stage of bvFTD (CDR 0.5), patients exhibited cognitive symptoms, including reduced error sensitivity and slower response times, despite relatively preserved attention, memory, and cognitive control. Moreover, their language performance scores were low, which appeared to stem from inattention, slowed information processing, or lack of effort. Overall, at the mild disease stage (CDR 1), bvFTD patients showed better performance than AD patients on episodic memory and set-shifting tasks, but scored worse in lexical fluency, emotion naming, and error sensitivity (bvFTD individuals at CDR 0.5 made more errors than AD group at CDR 2; Ranasinghe et al., 2016). These results suggest that bvFTD patients’ poor performance is not always due to cognitive deficits but rather to non-cooperation with the test procedure, since these patients show an early amotivational syndrome that reduces their attention and concern about accuracy. Moreover, the increased tendency of bvFTD patients to rule violations contributes to neuropsychological scores since the mild disease stage (Ranasinghe et al., 2016).

## 5 Decision-making tasks in patients with frontal lesions

Lesions to the prefrontal cortex (PFC) are strongly correlated with impairments in decision-making, particularly in tasks involving risk assessment, value-based choices, and cognitive control (Szczepanski and Knight, 2014). Bechara and colleagues in 1994, finely-tuned the gambling task to resemble real-life decision-making in patients with severe damage to PFC (Bechara et al., 1994). Patients with lesioned ventromedial PFC (VMPFC) consistently failed to choose advantageously even after repeated losses, revealing a deep-seated cognitive dysfunction. This study proposed three different possibilities to justify the patients’ behavior. Firstly, they could be particularly sensitive to reward; secondly, they could be insensitive to punishment, so that the probability of obtaining a reward could always be more attractive. Finally, it could be possible that patients were insensitive to future consequences, regardless of whether they were positive or negative (Bechara et al., 1994). To determine which possibility was more plausible, a modified version of the gambling task was developed. In this version, the order of punishment and reward was reversed, with the first one becoming immediate while the second one was delayed. Even through the modified version of the task, control individuals were able to shift their preference toward good decks during the task performance, while patients continued to favor disadvantageous options, displaying a decision-making pattern nearly identical to their performance on the original task. Thence, it was possible to conclude that patients are neither reward-driven nor punishment-blind, but rather, they are insensitive to future consequences. Furthermore, not even a worsening of future consequences could shift the behavior of VMPFC lesion patients toward more advantageous choices. This was also proven by an additional version of the gambling task, which consisted of an increased delayed punishment for the original version and a decreased delayed reward for the modified one. These results supported the fact that patients with VMPFC lesion exhibited the so-called “myopia for the future,” which persisted even when the severity of consequences increased, making them more prone to follow immediate prospects (Bechara et al., 2000). Further studies expanded these findings to assess if the amygdala damage could interfere with the decision-making process and whether it plays a distinct or similar role compared to lesions in the VMPFC. The results revealed that patients with amygdala lesions showed similarly disadvantageous behaviors, indicating a role for this structure in emotional learning and risk evaluation (Box 1; Bechara et al., 1999).

Box 1 **Skin conductance responses and decision-making**

It is important to underline that the impaired behavior of patients with VMPFC and amygdala lesions, is associated with their inability to generate anticipatory skin conductance responses (SCRs). Already in 1996, Bechara and colleagues decided to analyze SCRs of prefrontal patients and controls during the gambling task, as a measure of somatic state activation Indeed, while controls normally generate anticipatory SCRs when pondering risky choices, prefrontal patients failed to do so, and an altered passive avoidance learning is likely to be involved, since prefrontal lesions determine the inability to learn from previous mistakes and the tendency to continuously choosing disadvantageously (Bechara et al., 1996).

However, when focusing on reward or punishment SCRs, VMPFC lesioned patients were able to generate them after the selection of a specific card. Contrarily, amygdala damaged patients showed a strong impairment in the generation of reward or punishment SCRs, suggesting that VMPFC and amygdala damages affect in different ways the ability to generate these responses (Bechara et al., 1999).

Manes et al. (2002) decided to restrict their study to specific subregions of the PFC, comparing healthy controls with patients affected by discrete orbitofrontal (OBF) lesions, dorsolateral (DL) lesions, dorsomedial (DM) lesions, and large frontal lesions (Large; Manes et al., 2002).

All these groups of patients performed the IGT, and interestingly, only the OBF group showed a similar behavior to controls. Contrariwise, the DL group, the DM group, and the Large group made more disadvantageous choices, reflecting what was reported by previous studies on VMPFC lesion patients (Manes et al., 2002).

These same patient groups were also assessed with two additional tasks, the Gamble Task and the Risk Task. While overall decision quality was not significantly different across groups in the Gamble Task, the Large lesion group tended to place higher bets, showing more risk-taking behavior that resembled what is typically observed bvFTD patients (Rahman et al., 1999). In the Risk Task, patients in the Large and DL groups were less likely to choose the most probable outcome, unlike those in the OBF and DM groups, who performed more like controls. Taken together, these results show that only the group with extensive frontal damage displayed impairments across all three tasks: they made riskier decisions on the IGT, placed higher bets in the Gamble Task, and frequently chose the less likely but more rewarding option in the Risk Task. Conversely, individuals with pure orbitofrontal damage performed normally on all measures. This might seem to contradict earlier findings (e.g., Bechara et al., 1994), which emphasized orbitofrontal dysfunction. However, Manes et al. (2002) suggest that patients in earlier studies may have had broader lesions that extended beyond the OBF. In their study, careful lesion mapping helped to isolate the specific contributions of different frontal regions (Manes et al., 2002).

This neural framework is vividly mirrored in the real-life case of a 46-year-old man with no prior legal or psychiatric history, whose life was overtaken by symptoms of bvFTD. He began displaying personality changes, social withdrawal, and inappropriate behavior. At first, clinicians suspected a maniac episode or psychotic disorder. But detailed neuropsychological testing, along with reinterpretation of imaging, confirmed mild right frontal atrophy, frontal hypometabolism, and dopaminergic dysfunction all consistent with a diagnosis of bvFTD. Despite acknowledging the illegality of his actions, he lacked the emotional insight and inhibitory control to stop them (Karcher et al., 2024).

## 6 Brain anatomical correlates of compromised decision-making in FTD patients

Most studies investigating decision-making deficits using neuroimaging techniques have identified various patterns of brain atrophy and structural damage as key contributors to these impairments (Kipps et al., 2008).

Kloeters et al. (2013), in order to correlate the IGT performance with gray matter atrophy, examined bvFTD patients and controls with imaging acquisition and voxel-based morphometry (VBM). They hypothesized that the performance would be associated with atrophy in the VMPFC, a region consistently implicated in value-based decision-making. However, according to the findings, the VMPFC dysfunction alone did not fully account for the impaired IGT performance, therefore, they reanalyzed the IGT results applying a new scoring (proven to be a more sensitive metric) which emphasized learning and decision patterns over time (Kloeters et al., 2013).

Performance according to the modified IGT score was associated with atrophy not only in the prefrontal cortex but also in the occipital cortex and cerebellum. The involvement of the cerebellum, conventionally viewed as a motor control region, may reflect its role in higher-order cognitive functions, such as cognitive control and error monitoring, or may be linked to the visuomotor demands of the IGT, based on real-time coordination between visual feedback and motor responses.

This broader pattern of atrophy suggested that deficits in decision-making among bvFTD patients arise from disruptions across a neural network, rather than being localized to the VMPFC (Kloeters et al., 2013).

Furthermore, to investigate the bases of the impaired decision-making behavior observed in their case report, Nakaaki et al. (2007) performed a brain magnetic resonance imaging and a brain ^99m^Tc-ethylcysteinate dimer single-photon emission computed tomography (^99m^Tc-ECD-SPECT) examination. The former showed bilateral mild frontal lobe atrophy, while the latter revealed the presence of a bilateral hypoperfusion in the frontal lobes. More in detail, the left inferior frontal region (Brodmann area, BA 47), bilateral orbitofrontal and medial frontal regions (BA 9, 11), the left cingulate gyri (BA 32), and the left insula (BA 13) were characterized by a significant hypometabolism. These observations further supported the diagnosis of frontotemporal lobar degeneration. Moreover, since hypoperfusion was detected in both the orbital frontal and the medial frontal lobe in a patient with bilateral hypoperfusion, they suggested that both regions are necessary for decision-making (Nakaaki et al., 2007).

Additionally, when Spotorno et al. (2015) investigated bvFTD patients showing decision-making difficulties in a linguistic context characterized by ambiguity with high-resolution volumetric T1-weighted MRIs, reported a significant atrophy in medial and orbital frontal regions and in the right insula when compared to healthy seniors (Spotorno et al., 2015).

Since neurodegenerative dementia symptoms arise from specific and selective vulnerability of intrinsically connected networks (ICNs) in the human brain (Seeley et al., 2009), it becomes crucial to adopt a comprehensive, network-based perspective to understand the etiology of associated cognitive and behavioral impairments. In bvFTD, the most vulnerable and susceptible ICNs reported in literature are the Salience Network (SN; Seeley et al., 2009), the Semantic Appraisal Network (SAN; Ranasinghe et al., 2016; Seeley et al., 2009) and the Default Mode Network (DMN; Caminiti et al., 2015; Wong et al., 2018; Wilson et al., 2020). The SN, also known as cinguloinsular ICN, undergoes neurodegeneration in bvFTD and causes socioemotional symptoms (Seeley et al., 2009; Van Den Stock et al., 2019). It includes the ventral anterior insula, the anterior cingulate (ACC), the dorsomedial thalamus, hypothalamus, amygdala and periaqueductal gray (PAG; D. C. Perry et al., 2017). This brain network is involved in alerting the individual to endogenous or exogenous stimuli which are relevant for survival and reward, and it provides awareness, visceral emotional experiences, hedonic reward evaluations, and negative reinforcers (Sturm et al., 2018). Functional connectivity MRI (fcMRI) approach has revealed important differences in SN connectivity of bvFTD, svPPA, nfvPPA, and AD patients, resulting in a more effective approach than atrophy-based structural models (Toller et al., 2019). SN has been shown to be involved in spontaneous responses to socioemotional stimuli, and insula damage or dysfunction in bvFTD and svPPA cause disorganized attentional mechanisms during social cognition, changes in facial expressiveness responses, lack of emotional engagement and dysfunctions of emotional suppression mechanisms, thus resulting in emotion deficits (Kumfor et al., 2019; Marshall et al., 2019; Chen et al., 2020). Amygdala has also been studied in emotional attention mechanisms, which are disrupted in bvFTD, specifically relating to the ability of reading facial emotions (Bora et al., 2016).

While for bvFTD patients’ emotion reading dysfunction is mostly related to vulnerable SN, in svPPA this mechanism is susceptible to SAN, or limbic network, vulnerability (Seeley et al., 2009). SAN is described as the “prejudice network” or “affiliation network” since it is involved in automated evaluations and bias, and it provides hedonic evaluations of positive or negative valence in response to socioemotional and non-social stimuli (Eckart et al., 2012). Anatomically, the SAN includes the dorsomedial ATL, the subgenual cingulate area of the ventromedial OBF cortex, the head of caudate and nucleus accumbens, and the amygdala (Yang et al., 2021). In svPPA, reduced temporal-OBF cortex connection leads to inability to distinguish among the same valence emotions, such as sadness and fear (Hutchings et al., 2018). Damages in this connection have been identified as the key contributor to emotion recognition deficit in bvFTD (Multani et al., 2019). Interestingly, even ALS patients with damage in temporal-OBF cortex connection, but without cognitive impairments, showed inaccurate emotion attribution abilities (Crespi et al., 2016). Furthermore, the functional integrity of the ATL is important for contextualizing the emotions within a precise socioemotional semantic framework that improves empathic accuracy (Marshall et al., 2019). Accordingly, it has been reported that bvFTD patients with reduced ATL volume and connectivity displayed impaired ability to understand social norms and to recognize emotions such as humor or sarcasm (Multani et al., 2019). Moreover, bvFTD and svPPA patients’ hedonic evaluation abilities and prosocial motivation have been linked to the volume changes in the nucleus accumbens, which is involved in reward processing (Foster et al., 2022). Finally, the DMN, or memory network, has been identified to be vulnerable in AD (Seeley et al., 2009) but relatively preserved in bvFTD patients (Caminiti et al., 2015). This ICN is functionally divided into a ventral subsystem (which includes hippocampus, ventral posterior cingulate, posterior inferior parietal lobule) involved in retrieving and re-experiencing episodic memories, and a dorsal subsystem (including the antero-dorsal medial PFC, temporoparietal junction, posterior cingulate, precuneus) involved in selection and comparison of memories (Andrews-Hanna et al., 2010). Specifically, DMN is involved in interpreting individual’s own and others’ behaviors in specific contexts, through self-referential processing and social perspective taking, drawing on past experiences and memories. The ventral and dorsal DMN subsystems are important for advanced reasoning skills such as ToM and moral reasoning, allowing individuals to identify others’ goals and anticipate the social and emotional consequences of their actions (Caminiti et al., 2015). Interestingly, Kumfor et al. (2015) revealed that bvFTD patients display preserved DMN-related social contextualization function because when they were asked to read facial emotions combined with misleading contextual cues, they were not able to recognize emotions correctly, as they paid attention more to the context. This finding highlights the loss of SN and SAN functions related to emotion reading but preserved DMN contextual processing. Caminiti et al. (2015) also confirmed that DMN impaired connectivity in bvFTD patients negatively influenced their ability to accurately understand and attribute emotions to characters in a story-based empathy task (Caminiti et al., 2015).

## 7 Conclusion

FTD, particularly the behavioral variant, presents a profound disruption in decision-making processes, driven by progressive neurodegeneration in key brain regions that regulate cognitive control, social reasoning, and emotional evaluation (Elfgren et al., 1993; Gustafson, 1993; Neary et al., 1988; Cosseddu et al., 2020). This review explored decision-making impairments in FTD patients and the brain regions involved. Across multiple studies and neuropsychological assessments such as the IGT, BART, ACE and Moral Dilemma paradigms, bvFTD patients consistently display a preference for risky or impulsive choices, insensitivity to future consequences, and blunted emotional response to moral violations (Bechara et al., 1994; Lejuez et al., 2002; Mendez et al., 2005b; Mendez et al., 2005a; Kloeters et al., 2013). Furthermore, deficits in decision-making are not confined to abstract tasks but also emerge in real-world behaviors such as pathological gambling, financial misjudgments, and socially inappropriate conduct (Moheb et al., 2019). The clinical relevance of these impairments is emphasized by their direct impact on patient autonomy, caregiver burden, legal accountability, and quality of life. Notably, these deficits persist even in patients who performed within normal ranges on traditional cognitive tests, revealing that decision-making impairments may be among the earliest and most distinct markers of the disease (Mendez et al., 2005a). Recent studies emphasize the role of large-scale brain network vulnerability, particularly the SN, the SAN and the DMN in the cognitive and behavioral symptoms of FTD. The SN and the SAN dysfunction lead to impaired emotion processing and social behavior, while partial DMN preservation may support residual social contextual understanding in early bvFTD. Additionally, the early amotivation and rule violations of bvFTD patients can complicate tests interpretation. A range of neuropsychological tools (e.g., SEA, Mini-SEA, IGT, MBI, TASIT) and behavioral inventories (e.g., CBI, FBI, IRI, r-SMS) are exploited to detect these deficits, with IGT and moral dilemmas being particularly effective. Combining these tools with neuroimaging offers deeper insight into the neural basis of decision-making impairments in FTD. Neuroimaging data have significantly advanced our understanding of these impairments, revealing that decision-making dysfunction is not solely tied to the VMPFC but also involves broader atrophy patterns affecting the orbitofrontal cortex, anterior cingulate cortex, insula, occipital regions, and even the cerebellum (Kipps et al., 2008). These findings underscore the distributed nature of the neural networks involved in value-based decision-making, moral judgment, and risk assessment. We sought to provide a comprehensive overview of decision-making impairments in FTD, integrating neuropsychological, behavioral, and neuroimaging evidence within a network-based framework. This narrative review highlights the clinical relevance of several diagnostic tools and emphasizes the early detection of deficits, even early stages of FTD progression. However, it lacks recent studies focusing on the refinement of neuropsychological assessments to measure the impaired decision-making behavior in FTD patients. Moreover, the present study highlights the most commonly used tests for assessing decision-making, but the correct test selection should be guided by the clinician and adapted to the disease stage to accurately capture relevant neuropsychological aspects.

## Glossary

ACE: Addenbrooke’s Cognitive Examination
AD: Alzheimer’s disease
ATL: Anterior Temporal Lobes
BART: Balloon Analog Risk Task
bvFTD: behavioral variant of Frontotemporal dementia
CBI: Cambridge Behavioral Inventory
CDR: Clinical Dementia Rating
CS**-SC**: Controlled Social-Semantic Cognition
DART: Dynamic Affect Recognition Test
DL: Dorsolateral
DM: Dorsomedial
DMN: Default Mode Network
ESCB: Executive and Social Cognition Battery
FBI: Frontal Behavioral Inventory
FTD: Frontotemporal dementia
fvFTD: frontal variant of Frontotemporal dementia
hiACE: High-functioning Addenbrooke’s Cognitive Examination
ICNs: Intrinsically Connected Networks
IGT: Iowa Gambling Task
INP: Impaired Neuropsychological Performance
IRI**-EC**: Interpersonal Reactivity Index – Empathic Concern
IRI**-PT**: Interpersonal Reactivity Index – Perspective Taking
Large: Large Frontal Involvement
loACE: Low-functioning Addenbrooke’s Cognitive Examination
MBI: Moral Behavior Inventory
nfvPPA: Nonfluent-Variant PPA
NNP: Normal Neuropsychological Performance
NPI: Neuropsychiatric Inventory
OBF: Orbitofrontal
PPA: Primary Progressive Aphasia PPA
r**-SMS**: Revised Self-Monitoring Scale
rtvFTD: Right temporal variant FTD
SAN: Semantic Appraisal Network
SEA: Social and Emotional Assessment
svPPA: Semantic-Variant PPA
TASIT: The Awareness of Social Inference Test
TOM: Theory of Mind
UDS**-FTLD**: Uniform Data Set – Frontotemporal Lobar Degeneration
VBM: Voxel-Based Morphometry
VM: Ventromedial
VMPFC: Ventromedial Prefrontal Cortex
